# Supplementary material for: A computational systems approach identifies synergistic specification genes that facilitate lineage conversion to prostate tissue
Source: Nat Commun. 2017 Apr 21;8:14662. doi: 10.1038/ncomms14662 (PMC5413950; doi:10.1038/ncomms14662)
Supplement: Supplementary Information — Supplementary Figures, Supplementary Tables. [file ncomms14662-s1.pdf]

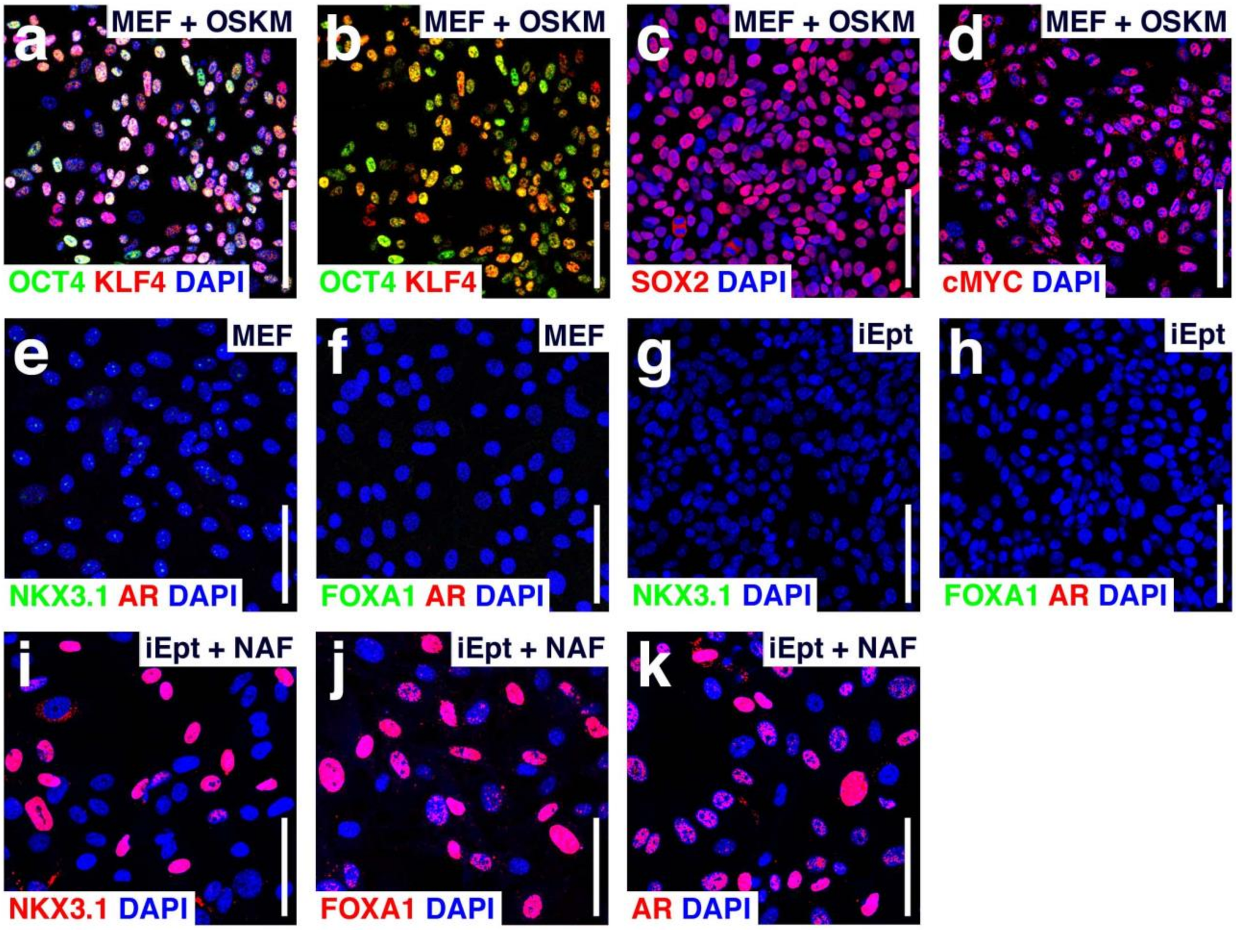

Supplementary Figure 1. Expression of OSKM, Nkx3.1, Foxa1, and AR. a-d, Expression of exogenously expressed OCT4 and KLF (a), SOX2 (b), and cMYC (d) in MEFs infected with retroviruses expressing OSKM; scale bars correspond to 50 microns. The infection efficiencies for each individual factor were quantitated (n=4 independent experiments) as 85.2% ± 5.9% for OCT4, 84.6% ± 6.6% for SOX2, 84.4 ± 2.9% for KLF4, and 90.8% ± 6.4% for cMYC; 82.4% ± 1.7% of the cells co-expressed OCT4 and KLF4. Based upon these observed efficiencies for expression of the single factors and for OCT4 and KLF4 co-expression, we can calculate an estimated minimal percentage of cells co-expressing all four factors as 43.1%, and an estimated maximal percentage of cells co-expressing all four factors as 84.1% (minimal percentage = SOX2% + cMyc% + OCT4/KLF4% – 200; maximal percentage = min(SOX2%, cMYC%, OCT4/KLF4%)). **e-h,** Nkx3.1, Foxa1, and AR are not detected by immunostaining in MEFs (e,f) or iEpt cells (g,h). **i-k,** iEpt cells infected with lentiviruses expressing all three MRs (Nkx3.1, Foxa1, and AR) show high levels of expression of each MR at 4 days post-infection.

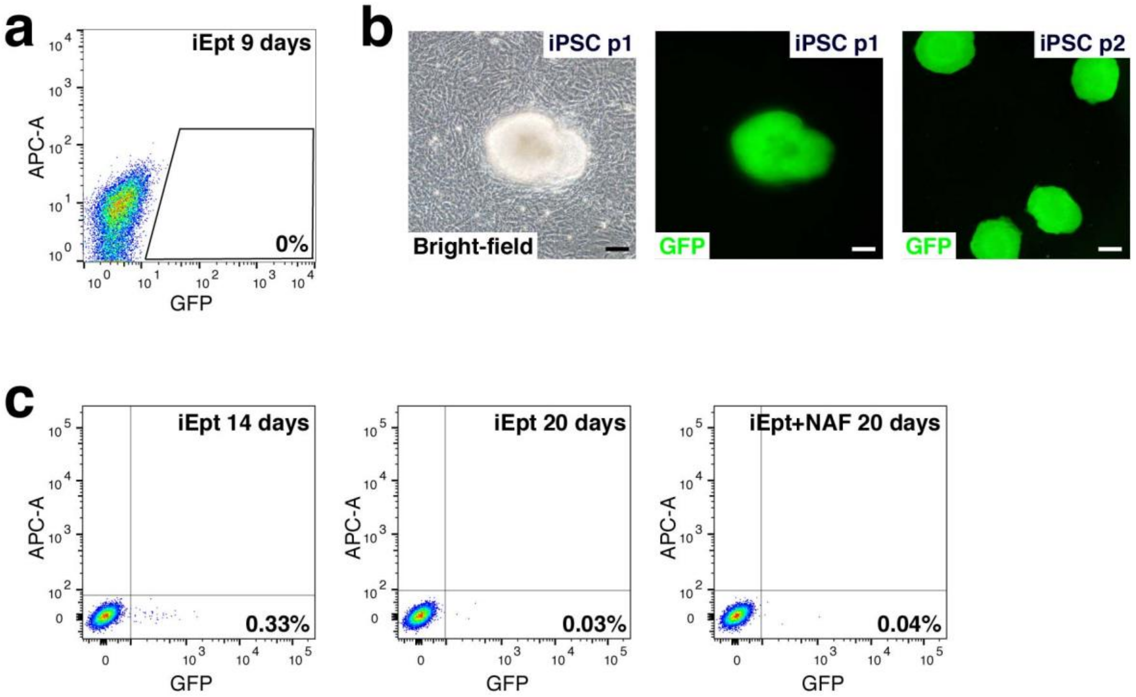

Supplementary Figure 2. Analysis of endogenous *Oct4* expression during primed conversion. a, iEpt cells generated from *Oct4-GFP* knock-in MEFs lack GFP-positive cells at 9 days after OSKM expression. Representative data are shown from 2 independent experiments. **b**, Efficient formation of iPSC colonies from *Oct4-GFP* MEFs under iPSC culture conditions in the presence of LIF; scale bars correspond to 50 microns. **c**, iEpt cells generated from *Tg(Oct4-GFP)* MEFs have negligible GFP-positive cells at 14 and 20 days after OSKM expression, with similar results after infection with the NAF virus combination. Representative data are shown from 2 independent experiments.

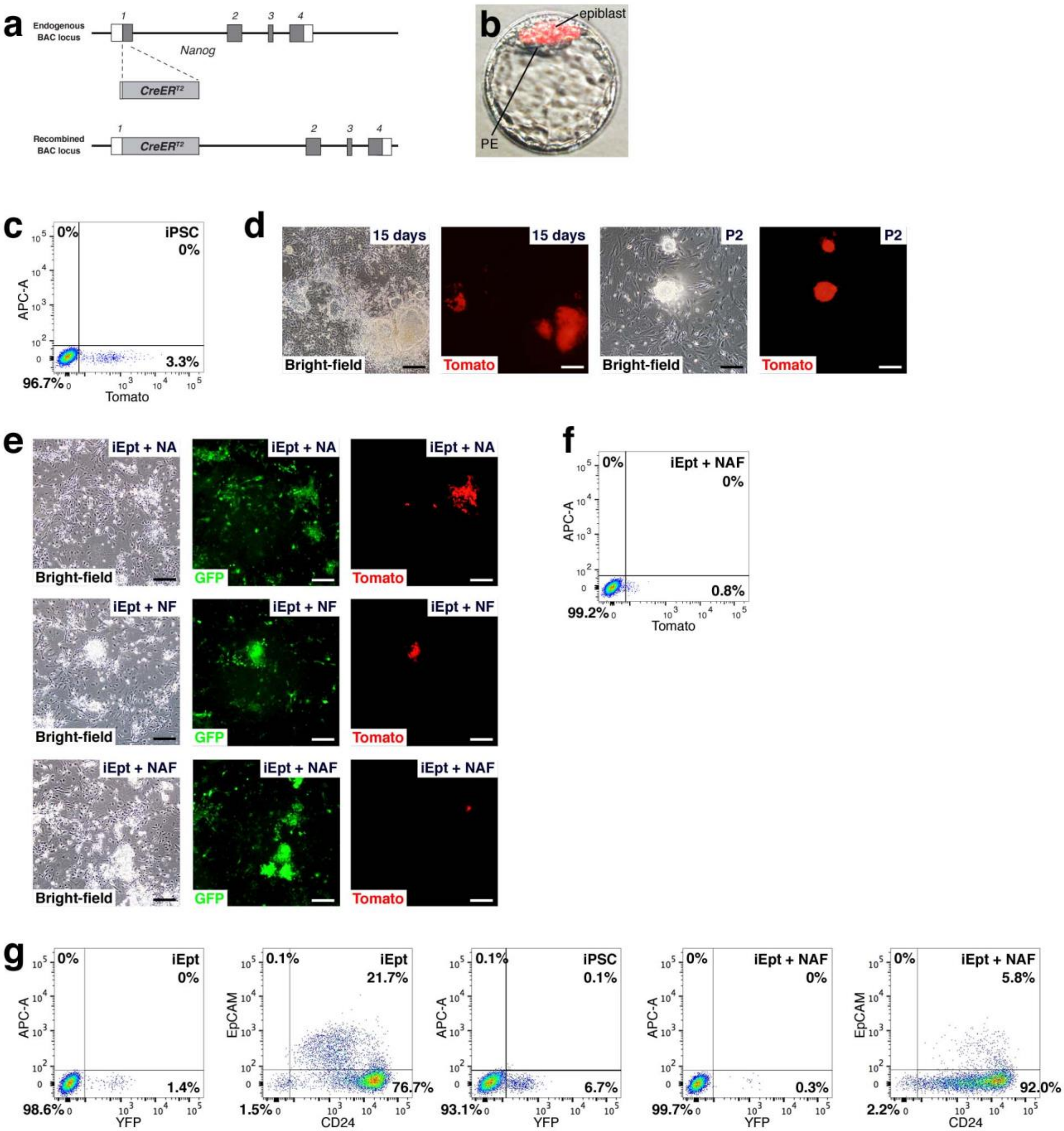

Supplementary Figure 3. Analysis of a transient pluripotent state during primed conversion. a, Schematic for generation of *Tg(Nanog-CreER^{T2})* mice. Recombineering was performed to insert a *CreER^{T2}* cassette precisely at the translation initiation site of *Nanog* in a bacterial artificial chromosome (BAC), followed by generation of transgenic mice. **b,** Expression of Tomato detected by direct visualization in *Tg(Nanog-CreER^{T2}); R26R-Tomato* blastocyst.

Embryos were collected at E3.5 and treated with 1 µM 4-OHT for 5 hrs before imaging. APC channel detects autofluorescence. **c**, At 15 days after infection of *Tg(Nanog-CreER^{T2}); R26R-Tomato* MEFs with OSKM retroviruses and culture in iPSC reprogramming conditions in the continual presence of 1 µM 4-OHT, 3.3% of cells are Tomato-positive (and hence passed through a Nanog-positive state). Representative data are shown from 4 independent experiments. **d**, Tomato-positive iPSC colonies derived from *Tg(Nanog-CreER^{T2}); R26R-Tomato* MEFs at 15 days after OSKM expression and at passage 2 (P2); scale bars indicate 100 microns. Representative images are shown from 2 independent experiments. **e**, iEpt cells expressing various MR combinations (NA, NF, and NAF) derived from *Tg(Nanog-CreER^{T2}); R26R-Tomato* MEFs display infrequent Tomato-positive cells when cultured in the continual presence of 1 µM 4-OHT; GFP is expressed by *Nkx3.1-IRES-GFP* infected cells; scale bars indicate 100 microns. Representative images are shown from 3 independent experiments. **f**, Similar to *Tg(Nanog-CreER^{T2}); R26R-Tomato* iEpt and iEpt+MR cells, iEpt+NAF cells derived from *Nanog-CreER/+: R26R-Tomato/+* MEFs accumulate a small percentage of Tomato-positive cells at 20 days after retroviral OSKM expression in basal epithelial media with continuous 4-OHT. Representative data are shown from 2 independent experiments. **g,** iEpt cells derived from *Oct4$^{CreER/+}$; R26R-YFP* mice accumulate a small percentage of YFP-positive cells in basal epithelial media with continuous 4-OHT at 25 days after OSKM expression, but at a higher percentage in iPSC culture conditions. The percentage of YFP-positive cells is lower in iEpt+NAF cultures, consistent with a reduction in the EpCAM^{+}/CD24^{+} population. Representative data are shown from 2 independent experiments.

Supplementary Table 1. Analysis of experimental synergy of master regulators of prostate organogenesis

**Additive effects**	**Synergy t-value**	**Synergy p-value**
**Foxa1** and **Nkx3.1**	3.5	0.020
**Foxa1** and **Nkx3.1** and **AR**	3.7	0.0023
pair(**Foxa1, Nkx3.1**) and **AR**	3.6	0.0027
pair(**Nkx3.1, AR**) and **Foxa1**	3.6	0.0028
pair(**Foxa1, AR**) and **Nkx3.1**	2.8	0.011

Predicted additive effects were estimated using a log-linear model based upon observed efficiencies of prostate tissue formation in the reprogramming assay. Comparison of the predicted additive effects to the observed (synergistic) effect was performed using a one-sample t-test.

Supplementary Table 2. Analysis of exogenous gene expression in tissue grafts

	Ratio of 3' UTR/coding region average read counts	
**Sample**	**Nkx3.1 (mouse)**	**Foxa1 (mouse)**
FS002 (iEpt+NAF)	2.22	4.32
FS003 (iEpt+NAF)	2.19	4.13
FS005 (iEpt+NAF)	1.99	3.83
FS007 (iEpt+NAF)	0	0
FS008 (iEpt+NAF)	0	0
Prostate (average)	3.39	4.95

	Coding region average read counts				
**Sample**	**AR**	**OCT4**	**SOX2**	**KLF4**	**MYC**
FS002 (iEpt+NAF)	0	29	76	40	26
FS003 (iEpt+NAF)	0	15	41	20	20
FS005 (iEpt+NAF)	0	128	117	62	24
FS007 (iEpt+NAF)	33	23	1040	497	226
FS008 (iEpt+NAF)	37	0	582	229	420
Prostate (average)	0	0	1	0	0

Expression of exogenous MRs and pluripotency factors was determined from average read counts based on RNA-seq data (see Methods) for the untranslated regions (UTRs) and coding regions. For exogenous mouse MRs (Nkx3.1 and Foxa1), the ratio of average read counts between 3' UTR and coding region is shown, with a higher ratio indicating the presence of endogenous transcripts, and a lower ratio indicating the presence of exogenous transcripts (that lack UTRs). For exogenous human AR and pluripotency factors (OSKM), which lack UTRs, the average read counts for the coding regions are shown, indicating the presence or absence of these transcripts. Read counts for six independent prostate samples were averaged and used for comparison.

Supplementary Table 3. Antibodies used in this study.

For immunofluorescence and immunohistochemistry			
**Antigen**	**Supplier**	**Ig type**	**Dilution**
AR	Sigma A9853	rabbit IgG	1:500
CK5	Covance PRB-160P	rabbit IgG	1:1000
CK5	Covance SIG-3475	chicken IgY	1:500
CK8	Abcam ab14053	chicken IgY	1:500
CK8	Developmental Studies Hybridoma Bank, clone TROMA-1	rat IgG2a	1:100
CK18	Abcam ab668, clone C-04	mouse IgG1	1:100
FoxA1	Abcam ab55178	mouse IgG2a	1:100
GFP	Abcam ab13970	chicken IgY	1:1000
Ki67	eBiosciences 14-5698, clone SolA15	rat IgG2a	1:1000
p63	Santa Cruz sc-8431	mouse IgG2a	1:200 - 1:600
p63	Santa Cruz sc-8343	rabbit IgG	1:100
Probasin	Santa Cruz sc-17124, clone M-18	goat IgG	1:100
Vimentin	Cell Signaling 5741S, D21H3	rabbit IgG	1:500
Smooth Muscle Actin	Sigma A 2547, clone 1A4	mouse IgG2a	1:1000
Beta-catenin	BD Transduction Labs 610154	mouse IgG1	1:1000
E-cadherin	BD Transduction Labs 610181, clone 36/E-Cadherin	mouse IgG2a	1:1000
Synaptophysin	BD Transduction Labs 611880	mouse IgG1	1:500
Nkx3.1	Axxora/AthenaES 0315	rabbit IgG	1:100

For flow cytometry		
**Antibody**	**Supplier**	**Dilution**
Anti-mouse CD326 (EPCAM) APC	BioLegend 118214	1:100
Anti-mouse CD326 (EpCAM) PE	eBioscience 12-5791	1: 300
Anti-mouse CD24 PerCP-eFluor® 710	eBioscience 46-0242	1:100
Anti-mouse CD24 PE-Cy7 antibody	eBioscience 25-0242	1:100
Anti-Mouse CD11b APC	eBioscience 17-0112	1:50
Anti-Mouse CD140a (PDGF Receptor a) PE	eBioscience 12-1401	1:100
Anti-mouse CD45 eFluor 450	eBioscience 48-0451	1:50
Anti-mouse CD31 eFluor 450	eBioscience 48-0311	1:50
Anti-mouse Ter119 eFluor 450	eBioscience 48-5921	1:50
